# Clinical comparison of radiofrequency ablation and surgical resection in treating pulmonar metastasis of hepatoblastoma

**DOI:** 10.3389/fonc.2025.1551799

**Published:** 2025-11-17

**Authors:** Yang Li, Hongxin Niu, Huirong Xu, Jian Wang, Jingfu Wang, Yizhi Liu, Xuena Liu

**Affiliations:** 1 Department of Pediatric Oncology, Affiliated Cancer Hospital of Shandong First Medical University, Jinan, Shandong, China; 2 Department of Interventional, Qilu Hospital (Qingdao), Cheeloo College of Medicine, Shandong University, Qingdao, Shandong, China; 3 Department of Interventional, Affiliated Cancer Hospital of Shandong First Medical University, Jinan, Shandong, China; 4 Department of Minimally Invasive Intervention, the Third Affiliated Hospital of Shandong First Medical University, Jinan, Shandong, China; 5 School of Public Health, Shandong First Medical University & Shandong Academy of Medical Sciences, Jinan, Shandong, China

**Keywords:** radiofrequency ablation, surgical resection, hepatoblastoma, pulmonary metastasis, children

## Abstract

**Objective:**

To explore the efficacy variances between radiofrequency ablation and traditional surgical resection in treating pulmonary metastasis of hepatoblastoma in children, and to provide more feasible methods for the treatment of this disease.

**Methods:**

A total of 91 pediatric hepatoblastoma (HB) patients with pulmonary metastases, admitted to Shandong Cancer Hospital between Sept. 2021 and Oct. 2023, were enrolled in this study. Among them, 31 received radiofrequency ablation for pulmonary metastasis eradication, while 60 underwent surgical resection to remove the pulmonary metastases. To assess the comparative effectiveness of these two interventional approaches in treating pediatric pulmonary metastases from hepatoblastoma, differences between the two groups were analyzed in terms of alpha-fetoprotein (AFP) levels, complication rates, antibiotic use, operative duration, length of hospital stay, and hospitalization costs.

**Results:**

The average levels of AFP in both the radiofrequency ablation group and the surgical resection group decreased significantly after treatment, with no statistical significance between the groups (*P*>0.05). The incidences of pneumonia, pulmonary atelectasis, and pneumothorax were statistically different between the groups (*P* < 0.05). Antibiotic use was markedly lower in the radiofrequency ablation group (χ^2^ = 43.4291, *P* < 0.001) as was the usage of advanced antibiotics (χ^2^ = 56.3477, *P* < 0.001) compared to the surgical group. Additionally, both the operation time (*t* = 11.186, *P* < 0.001) and hospitalization duration (*t* = 6.064, *P* < 0.001) were significantly shorter in the radiofrequency ablation group. The treatment costs for the radiofrequency ablation group were also significantly lower than those for the surgical group (*t* = 3.092, *P* = 0.003).

**Conclusion:**

Radiofrequency ablation for HB pulmonary metastases is more feasible, safer, and cost-effective due to its advantages of less invasive and painful, faster recovery, economical, and lower adjacent complication rate.

## Introduction

Liver tumors in children are uncommon, comprising approximately 5% to 6% of abdominal masses, which 2/3 being malignant, hepatoblastoma(RRID: MGI:5784899) is the predominant primary malignant liver tumor in this demographic (1, 2) presenting an incidence rate of about 1.5/1, 000, 000 (3). Notably, 90% of HB (RRID: MGI:5784899) cases occur in children younger than 3 years, with a male-to-female ratio ranging from 1.5:1 to 2.0:1 (4, 5). The etiology of HB(RRID: MGI:5784899) remains elusive, although factors such as premature birth, maternal age, infertility, smoking, obesity, hypertension(RRID: RRRC_00794), oligohydramnios, familial adenomatous polyposis, and Bwckwith-Wiedemann syndrome (BWS) are considered potential risk factors (6–8). HB’s clinical manifestations are typically nonspecific initially, often leading to incidental diagnosis due to a palpable right epigastric mass (9) Subsequent symptoms may include abdominal distension, pain, anorexia, nausea, vomiting, weight loss, dyspnea, fever, jaundice (RRID: IMSR_ORNL:59TNP), hepatic fibrosis (RRID: MGI:5637814) (10–12), and, in rare cases, peripubertal precocious puberty in males induced by human chorionic gonadotropin secretion (RRID: IMSR_JAX:006619) (13). HB(RRID: MGI:5784899) is notably aggressive, prone to widespread metastasis via hematogenous and lymphatic pathways(RRID: MMRRC_046273-MU), frequently affecting lymph nodes and the abdominal cavity (14), with the lungs being the most prevalent site for metastatic deposits (15–17).

Epidemiological studies have indicated that the prevalence of HB(RRID: MGI:5784899) is notably higher in Asians populations compared to European and American ones (5), though the reasons behind this disparity remain unclear. Over recent decades, the incidence of HB(RRID: MGI:5784899) in China has been progressively increasing (18). Consequently, identifying effective, minimally invasive, rapid recovery, and cost-efficient treatment modalities is essential for enhancing the survival rates of children afflicted with HB(RRID: MGI:5784899). Radiofrequency ablation (RFA), as an innovative treatment method, presents a promising alternative to conventional surgical lesion removal. With advancements in RFA technology and its proven therapeutic efficacy, both domestic and international medical communities have increasingly recognized its value. It should be made clear that, to date, RFA has not been established as the standard treatment for pulmonary metastases of hepatoblastoma (HB). Its application is usually limited to specific cases where surgical resection is not feasible due to factors such as the number, size or location of the metastases, and it is not a first-line alternative to surgical resection. While numerous clinical reports have documented RFA’s application in treating adult liver tumors, its use in pediatric hepatoblastoma (RRID: MGI:5784899), especially for treating pulmonary metastases, is less reported. Moreover, comprehensive studies comparing the overall treatment outcomes of RFA and surgical intervention for hepatoblastoma (RRID: MGI:5784899) pulmonary metastases are absent. This study, Therefore, aims to assess the therapeutic efficacy of radiofrequency ablation for HB (RRID: MGI: 5784899) pulmonary metastases by retrospectively analyzing the clinical outcomes and follow-up data of children who have undergone both surgical resection and radiofrequency ablation at our hospital.

## Materials and methods

### Study objects

This study included 91 children diagnosed with stage IV (with distant metastases to lungs and other organs) hepatoblastoma (RRID: MGI:5784899), featuring pulmonary metastases and metastases to other organs, treated at the Children’s Tumor Ward of Shandong Tumor Hospital from September 2021 to October 2023.

Children diagnosed with hepatoblastoma at the Pediatric Oncology Ward of Shandong Cancer Hospital from September 2021 to October 2023 were enrolled as the research subjects. In this study, the subjects were divided into the radiofrequency ablation group and the surgical resection group according to the surgical intervention methods for pulmonary metastases. To ensure the comparability between the two groups, uniform inclusion criteria were established for both groups, which are specified as follows:(1)Age range: 2–18 years, with no gender restrictions; (2)Confirmation of disease: Pathologically confirmed hepatoblastoma (RRID: MGI:5784899) (3); Clinical stage: Children with stage IV hepatoblastoma with pulmonary metastases at initial diagnosis (4); Characteristics of lesions: The number of unilateral pulmonary lesions ≤ 5, and the maximum diameter ≤ 3 cm (Note: This restriction is based on the consideration that repeated puncture and ablation may cause complications such as pulmonary hemorrhage and severe pneumonia; when the diameter of the lesion > 3 cm, it may lead to incomplete necrosis of the tumor edge, thereby affecting the treatment effect) (5); Location of lesions: Tumors confined to pulmonary tissue without breaching the pleura, and with a distance of > 5 mm from the heart, major blood vessels, and pleura (6); All subjects had previously received high-risk hepatoblastoma chemotherapy regimens mainly containing cisplatin and epirubicin, and had not received pulmonary radiotherapy.

### Treatment method

91 children received first-line chemotherapy for high-risk groups before lesion resection, accompanied by supportive treatments (antiemetic, gastric protection, hepatoprotection, myocardial nutrition) tailored to each child’s needs during chemotherapy.

**Figure 1:**
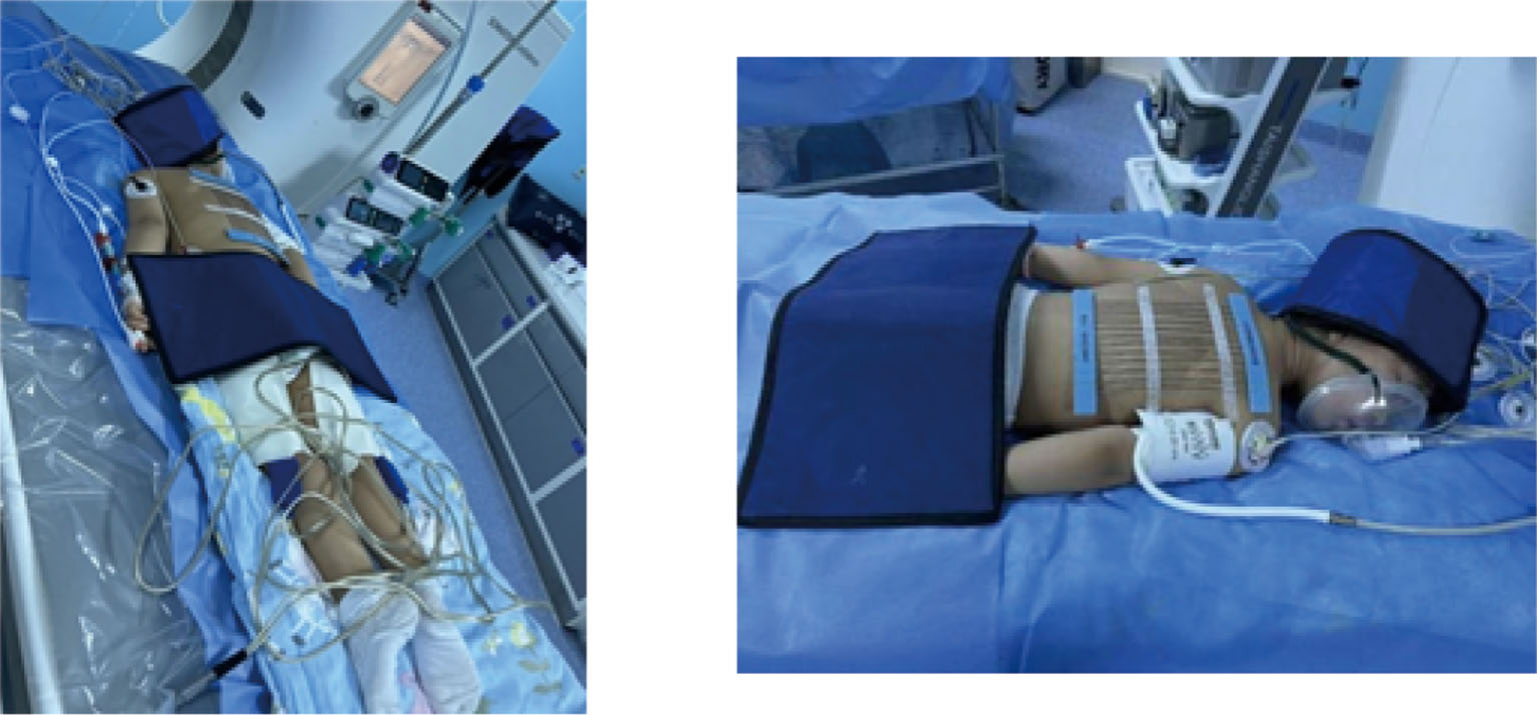
Preparation and radiological protection for ablating a pediatric hepatoblastoma under CT guidance and radiological protection.

**Figure 2:**
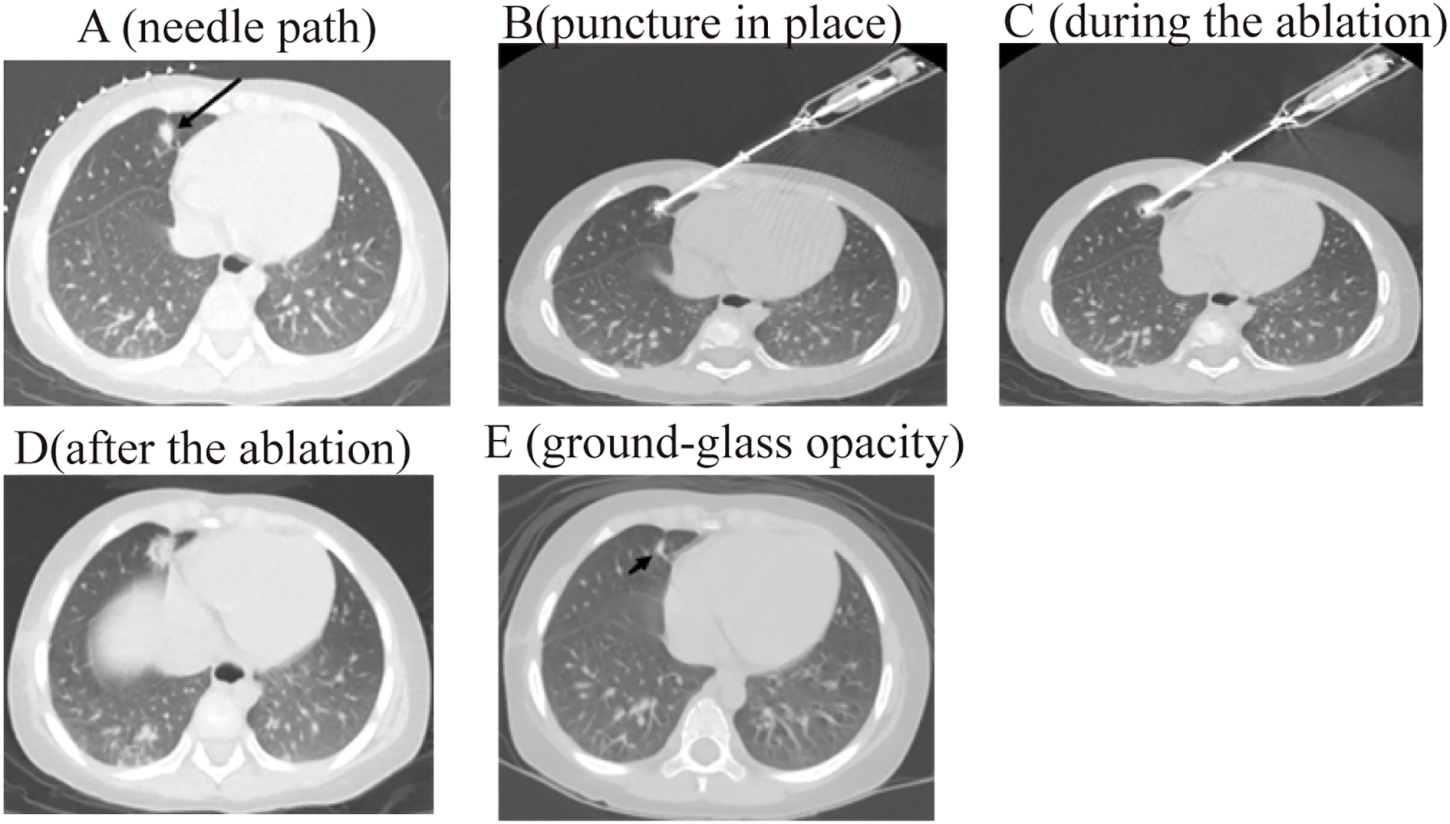
The process of radiofrequency ablation of pulmonary metastases of pediatric hepatoblastoma under CT guidance. .

The procedure for CT-guided radiofrequency ablation of pulmonary metastases proceeds as follows: The patient, a child, undergoes non-intubation general anesthesia using either propofol or sufentanil. Based on the lesion’s location as identified in preoperative CT (RRID: MGI: 2162669) scans, the patient is positioned on the CT (RRID: MGI:2162669) table to ensure accurate monitoring via electrocardiogram, oxygen delivery, and precise placement of the ablation needle’s electrode to expose the surgical site, while providing radiation protection to non-targeted areas (**Figure 1**). Markers are placed on the surgical area for precise identification of the ablation site and planning of the needle’s trajectory (**Figure 2A**). The skin at the intended entry point is disinfected and covered with a sterile drape before inserting a disposable radiofrequency ablation needle (model: RFDJ01-141712010, Lead Electron Corporation, Mianyang, China) through the designated puncture site to the exact target location, ensuring that the needle’s active tip extends 1–5 times the tumor’s margin (**Figure 2B**). Local anesthesia with 2% lidocaine is administered prior to needle insertion. The radiofrequency ablator (Model: LDRF-120S, Lead Electron Corporation, Mianyang, China) is then connected and activated under controlled conditions, with settings adjusted to 15–30 watts for 2–3 minutes, depending on the lesion’s size. Post-ablation, CT(RRID: MGI:2162669) imaging reveals a ground-glass opacity encompassing the lesion, indicating complete coverage (**Figure 2C**). A final CT (RRID: MGI: 2162669) scan confirms successful ablation and checks for any complications like pneumothorax or bleeding (**Figure 2D**).

The children in the surgical resection group underwent pulmonary wedge resection.

### Follow-up

Follow-up was conducted by tracking outpatient visits, inpatient departments, and regular home telephone follow-ups to monitor the survival status of the children and update their health indicators.

### Data collection

Detailed clinical data of the children were meticulously documented, encompassing gender, age, birth weight, date of diagnosis, clinical features, imaging features, surgery date, surgery duration, treatment costs, length of hospital stay, laboratory test results, antibiotic usage, complications, and disease outcomes.

Among them, the imaging assessment adopted the RECIST 1.1 standard as the main assessment method. During the specific operation process, two radiologists with over five years of experience in chest imaging diagnosis independently read the films, measuring and recording the longest diameter, short diameter of the lesion and related imaging features, etc. If the difference in the assessment results between the two exceeds 10%, a consensus conclusion is reached through joint film reading by two people to ensure the objectivity and repeatability of the assessment.

### Statistical methods

Data were compiled and analyzed using EXCEL and SPSS (RRID: SCR_002865) software. Quantitative data were presented as (mean ± standard deviation) or median (interquartile range), with group comparisons conducted using t-test or rank-sum tests. Qualitative data were represented by percentages, and comparisons between groups were performed using chi-square tests or Fisher’s exact probability test, considering *P* < 0.05 as indicative of a statistically significant difference.

### Data availability statement

The original data of this study were obtained from the Cancer Hospital Affiliated to Shandong First Medical University. All relevant derivative data supporting the results of this study can be obtained at the request of the corresponding author.Any other data generated in this study can be freely obtained in the article and its supplementary data.

## Results

### General information

From September 2021 to October 2023, a total of 290 children were diagnosed with hepatoblastoma in the Pediatric Oncology Ward of Shandong Cancer Hospital. Among them, 172 cases were complicated with pulmonary metastasis, and 105 children underwent surgical intervention (including pulmonary wedge resection or radiofrequency ablation of pulmonary lesions). A total of 91 children who met the inclusion criteria were enrolled in this study, with 31 cases in the radiofrequency ablation group and 60 cases in the surgical resection group. Fourteen children were excluded due to failure to meet the inclusion criteria: 1 child died within two months after surgery, 2 children were lost to follow-up, and 11 children were excluded because the number of resected nodules in the unilateral lung exceeded 5.

In the radiofrequency ablation group, there were 21 male cases and 10 female cases, with a male-to-female ratio of 2.1:1. In the surgical treatment group, there were 35 male cases and 25 female cases, resulting in a male-to-female ratio of 1.4:1; The ages in the radiofrequency ablation group ranged from 2 to 15 years, average (76.00 ± 42.541) months, while the surgical treatment group’s ages ranged from 1 year and 3 months to 21 years, with an average of (63.83 ± 44.721) months. Of the 91 patients, only 2 received bilateral treatment, while the remaining all underwent unilateral treatment. Statistical analysis showed that there were no statistically significant differences in gender, age, preoperative AFP (RRID: NSRRC_0028) levels, number of lesions, distribution of lesions and average lesion diameter between the two groups of patients (*P*>0.05), indicating good comparability (**Table 1**).

**Table 1:** Comparison of general data between the two groups.

Variables	Radiofrequency ablation group	Surgical treatment group	*T/χ²/z*	*P* value
Age (*x±s* )	76.00±42.541	63.83±44.721	1.250	0.215
Sex(n(%)) male female	21(67.74) 10(32.26)	35(58.33) 25(41.67)	0.764	0.382
Preoperative AFP value(ng/ml)(M (Q1, Q3))	450.00(25.375,923)	99.45(23.75,840.25)	-1.166	0.244
Number of lesions(M (Q1, Q3))	1.00(1.00.2.00)	2.00(1.00.2.00)	-1.852	0.064
Average diameter of the lesion(mm)(M (Q1, Q3))	1.50(1.18,2.30)	1.42(0.90,1.74)	-1.585	0.113

### Comparison of treatment effects between two groups

As shown in **Table 2**, the AFP (RRID: NSRRC_0028) levels in both the radiofrequency ablation group and the surgical treatment group decreased significantly after treatment. however, no statistically significant difference was observed (*P*>0.05).

**Table 2:** Comparison of AFP levels preoperative and 3 months post-treatment (M (Q_1,_ Q_3_)).

Grouping	Preoperative AFP	AFP three months after operation	*Z*	*P* value
Radiofrequency ablation group	450.00(25.375,923.00)	197.50(15.15,1792.00)	0.546	0.585
Surgical treatment group	99.45(23.75,840.25)	13.05(2.7525,495.25)	1.615	0.106

### Assessment of postoperative CT results

3 months post-operation, children in the study underwent a CT (RRID: MGI:2162669) scan for follow-up. In the radiofrequency ablation group, out of 28 cases, 26 showed complete lesion resolution with ground-glass opacities observed (**Figure 2E**), one case resolved with vacuole formation, and another case experienced a new lesion in the left pulmonary. Meanwhile, in the surgical treatment group, out of 60 cases, 58 demonstrated lesion disappearance with scarring in the treated areas. One case developed sepsis and died one month later, and one case of recurrence approximately 1 cm from the treated area. The rates of lesion resection or destruction in both treatment groups exceeded 95%.

The Disease Control Rate (DCR) within 3 months was calculated according to the RISIT 1.1 standard. The results showed that the DCR value of the radiofrequency ablation group was 96.43%, and that of the surgical resection group was 96.67%.

### Deaths and complications

In the radiofrequency ablation group, there were no deaths related to the treatment or other causes. However, the surgical group experienced two fatalities, one due to sepsis and another resulting from multiple system organ failure.

Postoperatively, six children (19.35%) in the radiofrequency ablation group developed pneumonia, a singular complication in this cohort. Conversely, the surgical treatment group saw postoperative complications in 58 cases (96.67%), with 45 patients (75.00%) experiencing multiple complications. These included 58 instances of pneumonia, 34 of pulmonary atelectasis, 32 of pneumothorax (9 cases of hemopneumothorax and 23 of liquid pneumothorax), 8 cases of hypoproteinemia, 6 cases of pleural effusion, 1 case of hyperkalemia, and 1 case of electrolyte imbalance. Notably, the rates of pneumonia, pulmonary atelectasis, and pneumothorax presented significant statistical differences between the groups (*P* < 0.05), as detailed in **Table 3**.

**Table 3:** Comparison of postoperative complication rates between two groups (n(%)).

Complications	Radiofrequency ablation group	Surgical treatment group	*χ^2^*	*P* value
pneumonia	6(19.35)	58(96.67)	58.547	<0.001
pulmonary atelectasis	0(0.00)	34(56.67)	28.045	<0.001
pneumothorax	0(0.00)	32(53.33)	25.501	<0.001
hypoproteinemia	0(0.00)	8(13.33)	4.532	0.0822
pleural effusion	0(0.00)	6(10.00)	3.319	0.1688
hyperkalemia	0(0.00)	1(1.67)	–	0.6593
electrolyte disorder	0(0.00)	1(1.67)	–	0.6593

### Other indicators

Antibiotics use was observed in all 60 cases (100.00%) in the surgical treatment group, compared to only 13 cases (41.94%) in the radiofrequency ablation group, showcasing a significant difference in antibiotic utilization rates between the two groups (χ^2^ = 43.4291, *P* < 0.001). This indicates a substantially lower rate of antibiotic use in the radiofrequency ablation group. Moreover, high-level antibiotics were administered in just 3 cases (9.68%) in the radiofrequency ablation group and 54 cases (90.00%) in the surgical treatment group, further emphasizing the lower usage of high-level antibiotics in the radiofrequency ablation group (χ^2^ = 56.3477, *P* < 0.001) (**Table 4**).

**Table 4:** Comparison of other indicators before and after treatment between the two groups.

Indicators	Radiofrequency ablation group	Surgical treatment group	*T/z/χ^2^*	*P* value
Antibiotic usage (n(%))	13(41.94)	60(100.00)	43.429	<0.001
Advanced antibiotic usage (n(%))	3(9.68)	54(90.00)	56.348	<0.001
Operation time (min)(M(Q1,Q3))	21(16,26)	80(60,105)	7.444	<0.001
Hospitalization time (day(M(Q1,Q3))	4(3,5)	8(7,9)	6.253	<0.001
Hospitalization expense (yuan) (*x±s* )	21538.62±7646.00	26028.38±5939.28	3.092	0.003

**Figure 3:**
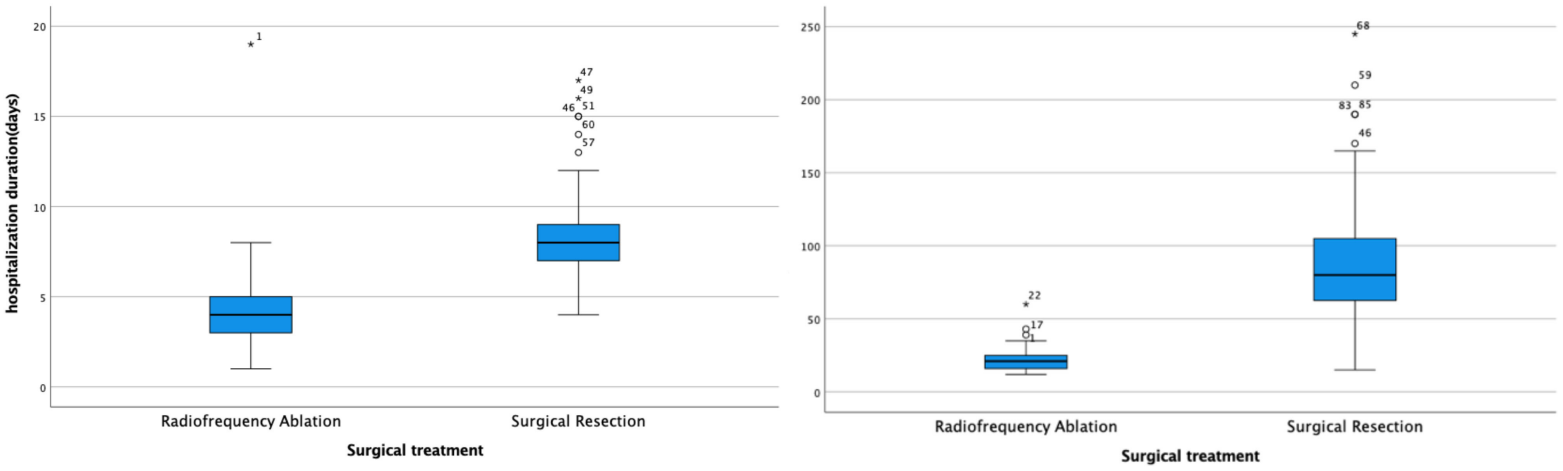
Box plots of hospitalization time and operation time in both groups.

Additionally, both the operation time (*t* = 11.186, *P* < 0.001) and hospitalization duration (*t* = 6.064, *P* < 0.001) were significantly shorter in the radiofrequency ablation group compared to the surgical treatment group (**Figure 3**).

The cost of treatment in the radiofrequency ablation group was significantly less than that in the surgical treatment group (*t* = 3.092, *P* = 0.003).

### Follow-up plan and current situation

Long-term oncological outcomes are of great significance for evaluating treatment regimens. Therefore, the research team has formulated a long-term follow-up plan: all enrolled patients will be included in a 5-year follow-up system. The follow-up time points include 3 months, 6 months, 1 year, 2 years, 3 years, and 5 years after the end of treatment. The key monitoring indicators include disease-free survival status, tumor recurrence, distant metastasis, long-term complications and mortality.

As of the last follow-up (December 2024), a total of 25 patients in the radiofrequency ablation group were followed up. Among them, the lesions in 24 patients disappeared, showing ground-glass opacity, and 1 patient had *in-situ* recurrence. In the surgical treatment group, 33 patients were followed up: the lesions in 28 patients disappeared with scars in the surgical area, 1 patient had recurrence within 1 cm, and 1 patient had distant recurrence.There were no deaths in either group.

In this study, 14 children who were not included in the study were also followed up, and relevant outcome data were collected, which are reported as follows (1): Tumor response: After receiving alternative treatments (such as other local ablation therapies or continued chemotherapy), 7 children achieved partial response (PR), 4 children had stable disease (SD), and 3 children had progressive disease (PD);(2) Survival status: As of the last follow-up, 7 children were still alive, 4 children died due to tumor progression or treatment-related complications, and 3 children were lost to follow-up;(3) Treatment-related complications: Among the 14 children, 14 cases had mild complications (such as low-grade fever and fatigue), 11 cases had moderate complications (such as pleural effusion requiring drainage), and no severe complications (such as severe pulmonary hemorrhage and multiple organ failure) were observed.

## Discussion

HB(RRID: MGI:5784899) is a prevalent solid abdominal tumor in children, arising from abnormal differentiation of epithelial hepatocyte (RRID: MGI:2177543) precursors during embryonic development (17, 19), It typically comprises embryonic epithelial tissues, cartilage or bone-like tissues, and embryonic mesenchymal tissues(RRID: MGI:2181551).

Currently, the treatment of HB (RRID: MGI:5784899) pulmonary metastasis is mainly based on chemotherapy combined with surgical resection. Although numerous scholars have reported that surgical resection yields definite therapeutic effects, its drawbacks such as significant trauma and a high incidence of complications remain unavoidable.

Radiofrequency ablation (RFA) utilizes radio waves within the 1k to 3MHz frequency range, emitted by an antenna, as a novel local therapy. This technique employs imaging technology to accurately target and position tumors. RFA represents a promising local therapy, employing the following mechanism: under imaging technology’s guidance for precise tumor targeting and positioning, the RF generator emits a current at 460kHz upon the RF needle’s entry into the lesion. This current, transformed into heat by the exposed electrode needle, generates rapid ionic vibration and friction in adjacent tissues, dispersing gradually to peripheral tissues. Consequently, local cells experience heat-induced coagulative necrosis and denaturation at a specific temperature threshold, effectuating tumor inactivation or localized radical treatment. Recent studies have indicated RFA’s capacity to bolster both local and systemic cellular immune responses (RRID: IMSR_GPT: T021709), facilitating the eradication of tumors and residual cells, and mitigating recurrence- a key factor in its noted long-term success and minimal recurrence rates. Although RFA is a well-established technique for adult tumors, its application in pediatric oncology is less common due to the small size of pediatric tumors, which necessitate heightened precision for target punctures within a 5 mm in diameter. To preserve the delicate pediatric pulmonary tissue, an 18G diameter ablation needle with a 10mm working end, causing minimal damage and yielding effective results, was chosen for this investigation.

In this study, the youngest participant was 1 year and 3 months old. Among all enrolled subjects, 83.1% were under 7 years of age, with those under 3 years accounting for 28.2%. Since all patients included in this study were diagnosed with stage IV hepatoblastoma with pulmonary metastases, most of the subjects had undergone an extended course of treatment after the onset of the disease. That is to say, the actual onset time of many children needs to be traced back by a certain period. Therefore, the age of the enrolled patients in this study was slightly older, which we consider to be normal.

Serum AFP(RRID:NSRRC_0028) levels can increase before changes are detectable through abdominal imaging in patients with hepatoblastoma. Consequently, AFP serves as a critical tumor marker for hepatoblastoma (HB), playing a vital role in both diagnosis and prognostic evaluation (20). Typically, HB develops between 6 months and 3 years of age, with the majority of cases showing AFP (RRID:NSRRC_0028) levels elevated by at least 80% to 90% above the norm. AFP(RRID:NSRRC_0028) levels below 100 ng/mL or above 1 million ng/mL indicate a high risk for adverse HB (RRID: MGI:5784899) outcomes (21, 22) In our study, serum AFP (RRID:NSRRC_0028) levels ranged from 1.92 to 60500 ng/ml. 38 children had AFP (RRID:NSRRC_0028) levels below 100 ng/mL, constituting 41.76% of the cases, with 10 in the radiofrequency ablation group and 28 in the surgical treatment group. No cases had AFP (RRID:NSRRC_0028) levels over 1 million ng/ml. Post-treatment, both groups saw a significant drop in AFP(RRID:NSRRC_0028) levels, with median levels reducing from 450.00 to 197.50 and 99.45 to 13.05, respectively, showing no significant statistical difference (*P*>0.05). This could be attributed to some children having multiple pulmonary metastatic lesions or developing new liver or other metastatic lesions post-treatment, although a significant AFP (RRID:NSRRC_0028) reduction was observed three months post-operatively, resulting in a broad range of AFP (RRID:NSRRC_0028) values. Future research may benefit from a stratified analysis of AFP (RRID: NSRRC_0028) levels shortly after surgery (e.g., 1–2 weeks) compared to later stages to derive more relevant insights.

3-month after surgery, CT (RRID: MGI:2162669) scans revealed a success rate exceeding 95% in the resection or destruction of pulmonary metastases for both treatment approaches, indicating that radiofrequency ablation’s efficacy is on par with traditional tumor “resection”.

The study revealed that the average duration of operations in the radiofrequency ablation (RFA) group was 21 minutes, significantly less than the 80 minutes observed in the surgical resection group. This difference underscores RFA’s efficiency in treating HB (RRID: MGI:5784899) pulmonary metastases by considerably reducing operation time, alleviating patient discomfort, and lowering the risk of intraoperative complications. Furthermore, postoperative follow-ups showed no deaths related to or unrelated to treatment in the RFA group, in contrast to two fatalities in the surgical treatment group. A notable disparity was observed in the incidence of postoperative complications between the groups (*P* < 0.05), with 6 (19.35%) instances of pneumonia as a solitary complication in the RFA group versus 58 cases (96.67%) of various complications in the surgical group, including pneumonia, pulmonary atelectasis, pneumothorax, hypoproteinemia, and pleural effusion, with 75% of these patients experiencing multiple complications. These findings suggest that RFA offers a safer and more viable alternative to surgical resection for the treatment of HB (RRID: MGI:5784899) pulmonary metastases.

The study also revealed that the radiofrequency ablation (RFA) group exhibited significantly lower usage of antibiotics, including high-level antibiotics, compared to the surgical resection group. In children, antibiotics can provoke stronger immune responses and a decreased ability to tolerate adverse effects compared to adults. Beyond common allergic reactions, toxic effects such as auditory nerve damage, dysfunction of the hematopoietic system, and renal, hepatic, and gastrointestinal damage are more intense and extensive. The most grave consequences include permanent alterations in immune function and the nervous system, particularly in children under three years old, whose organs and tissues are still developing and thus more vulnerable to lasting harm from antibiotics. Misuse of antibiotics or reliance on high-level antibiotics can increase the risk of secondary infections, which are harder and more perilous to manage. Additionally, extended antibiotic use may lead to resistance. The study demonstrated that the utilization of antibiotics, particularly high-level antibiotics, was significantly lower in the radiofrequency ablation group compared to the surgical treatment group. This finding suggests that radiofrequency ablation (RFA) can substantially reduce or even prevent the adverse effects associated with the use of antibiotics and high-level antibiotics in children.

Children with HB (RRID: MGI:5784899) represent a demographic that does not generate income and can inevitably impose psychological and economic strains on their families due to the prolonged nature of treatment, potentially increasing the risk of treatment discontinuation. The analysis results of hospitalization duration and costs between the two groups revealed that the average hospital stay was 4 days at a cost of 21, 538 yuan for the radiofrequency ablation group, compared to 8 days and 26, 028 yuan for the surgical treatment group. This demonstrates that both the duration and costs of hospitalization for the radiofrequency ablation group were significantly lower than those for the surgical treatment group. Consequently, radiofrequency ablation emerges as a more efficient and cost-effective treatment option relative to surgical resection.

In conclusion, both traditional surgical resection and radiofrequency ablation (RFA) offer viable local treatment options for HB (RRID: MGI:5784899) pulmonary metastasis in children, effectively controlling the lesion. However, CT-guided RFA emerges as a safe and efficacious innovative approach for treating HB (RRID: MGI:5784899) pulmonary metastases, characterized by precise localization, real-time monitoring, favorable outcomes, cost-effectiveness, minimal complications, reduced treatment duration, and greater patient acceptance. Given the advancements in pediatric RFA technology and equipment, the RFA technique holds significant promise and is recommended for broader adoption in HB (RRID: MGI:5784899) treatment.

This study is a single-center retrospective study and has certain limitations. (1) Despite the strict adherence to unified inclusion criteria in this study, non-random grouping may still introduce selection bias. For instance, non-random allocation could lead to differences in baseline physical conditions between the two groups (e.g., children with poorer physical fitness may be more inclined to receive radiofrequency ablation, while those with better physical fitness may prefer traditional surgical resection), thereby causing part of the difference in therapeutic effects to be affected by selection bias. Uncontrolled confounding factors may also interfere with the results (e.g., if children in the surgical resection group have underlying diseases, their high complication rate may be related to their baseline physical status). In addition, the limited sample size restricts the application of matching techniques and multivariate analysis. Future studies could optimize the design by expanding the sample size and adopting matching techniques to reduce the risk of Type II errors and the interference of confounding factors. (2) Single-center data are limited by the regional characteristics of patients and the diagnostic and therapeutic characteristics of the center, resulting in insufficient extrapolation of the results. To enhance the reliability and applicability of the conclusions, subsequent studies will adopt a multi-center prospective design to reduce bias and further explore the association between lesion characteristics and prognosis.

## Data Availability

The original contributions presented in the study are included in the article/supplementary material. Further inquiries can be directed to the corresponding author.
